# Molecular Analysis of Mixed Endometrioid and Serous Adenocarcinoma of the Endometrium

**DOI:** 10.1371/journal.pone.0130909

**Published:** 2015-07-01

**Authors:** Kate Lawrenson, Elham Pakzamir, Biao Liu, Janet M. Lee, Melissa K. Delgado, Kara Duncan, Simon A. Gayther, Song Liu, Lynda Roman, Paulette Mhawech-Fauceglia

**Affiliations:** 1 Department of Preventive Medicine, Keck School of Medicine, University of Southern California Norris Comprehensive Cancer Center, Los Angeles, California, 90033, United States of America; 2 Departments of Pathology, Keck School of Medicine, University of Southern California, Los Angeles, California, 90033, United States of America; 3 Department of Biostatistics and Bioinformatics, Roswell Park Cancer Institute, Buffalo, New York, 14263, United States of America; 4 Department of Gynecologic Oncology, University of Southern California, Los Angeles, California, 90033, United States of America; University of Quebec at Trois-Rivieres, CANADA

## Abstract

**Background:**

The molecular biology and cellular origins of mixed type endometrial carcinomas (MT-ECs) are poorly understood, and a Type II component of 10 percent or less may confer poorer prognoses.

**Methodology/Principal Findings:**

We studied 10 cases of MT-EC (containing endometrioid and serous differentiation), 5 pure low-grade endometrioid adenocarcinoma (EAC) and 5 pure uterine serous carcinoma (USC). Endometrioid and serous components of the MT-ECs were macrodissected and the expression of 60 candidate genes compared between MT-EC, pure USC and pure EAC. We found that four genes were differentially expressed when MT-ECs were compared to pure low-grade EAC: *CDKN2A* (P = 0.006), *H19* (P = 0.010), *HOMER2* (P = 0.009) and *TNNT1* (P = 0.006). Also while we found that even though MT-ECs closely resembled the molecular profiles of pure USCs, they also exhibit lower expression of *PAX8* compared to all pure cases combined (P = 0.035).

**Conclusion:**

Our data suggest that MT-EC exhibits the closest molecular and epidemiological similarities to pure USC and supports clinical observations that suggest patients with MT-EC should receive the same treatment as patients with pure serous carcinoma. Novel specific markers of MT-EC could be of diagnostic utility and could represent novel therapeutic targets in the future.

## Introduction

Endometrial cancers (ECs) are common gynecologic maligancies, accounting for 3.2% of all cancers in the US, and occurrence rates are steadily rising by ~1% each year [1]. ECs are classified as Type I or Type II. Type I tumors are mostly of endometrioid histology and present as low-grade, early-stage tumors (FIGO I and II) with favorable outcomes. Meanwhile Type II carcinomas, which represent less than 10% of total EC cases, typically have serous or clear cell histologies and are usually high-grade tumors with myometrial and lymphovascular invasion and an aggressive clinical course. Finally, mixed type endometrial carcinomas (MT-ECs) are tumors with both Type I and II components that represent ~10% of ECs. MT-ECs are rare tumors that are difficult to diagnose and, as a consequence, current occurrence estimates are probably inaccurate. There is no consensus among gynecologic oncologists about the best approach to the management and treatment of patients with mixed tumors. Historically, early-stage endometrial carcinomas with a Type II component of >25% of tumor volume are thought to behave as high-grade tumors and should be managed as such, by treating with chemo/radiation therapy [2]. More recent reports suggest that patients with early-stage mixed tumors with a Type II component even less than 10% had worse prognoses than pure endometrioid adenocarcinoma (EAC) cases and should therefore also be treated as high-grade tumors [3]. In a retrospective study by Fader et al, all patients with a uterine serous carcinoma (USC) component within their tumor specimens were found to be at a significant risk of recurrence and poor survival [4]. However, these conclusions were solely based on retrospective population studies and have not yet be corroborated by molecular studies.

Improving clinical diagnoses of MT-ECs is essential, as prognoses and treatments are markedly different for Type I and Type II tumors. However, reproducibility of diagnoses of cell type within ECs and particularly diagnosis of MT-EC is poor; in a recent study comparing the reproducibility of diagnoses of high-grade endometrial carcinoma across three different pathologists, there were major disagreements in over one-third (35.8%) of cases [5]. For MT-ECs there was frequent disagreement about the histology of the major component, but even more critically, there were also disagreements about whether a high-grade component was present or not. Furthermore, in a second report, the diagnosis was changed in 34% of endometrial primaries where 18% necessicated alteration of the management and most of the diagnostic changes pertained to tumor grade and subtype [6]. More often than not the currently available immunohistochemical markers are only partly informative, or even contradictory [5, 7]. It is clear that better molecular markers are needed that can be used by pathologists to accurately diagnose the presence of high-grade components in MT-ECs, and that diagnosis based on morphological criteria alone is not reliable or reproducible.

Improved detection of MT-EC will have a substantial impact on patient care and management as the consequences of misdiagnosis results in under- or over-treatment; both scenarios have serious consequences for patients and are associated with increased morbidity and mortality. In the case of early-stage MT-EC where the serous component is missed, the patients will be treated for a less aggressive Type I tumor and they will undergo total hysterectomy+bilateral salpingo-oophorectomy (TH+BSO) with or without radiation therapy. Without an aggressive treatment incorporating chemotherapy, the Type II component is more likely to recur and the tumor would be almost never slaveageable. Meanwhile, if the Type II component is over diagnosed, the patients will undergo TH+BSO followed by radiation/chemotherapy. The unnecessary chemotherapy is associated with high morbidity and mortality rates, and a myriad of severe side effects.

The biology and cellular origins of MT-EC are poorly understood. Whether the heterogeneous components within a mixed tumor represent an intermediate biology, or whether MT-ECs represent a Type II tumor masquerading as a Type I tumor (or *vice versa*) is not yet understood. In this current study we used molecular analysis of macrodissected MT-ECs to address this question and to characterize the molecular profiles of this tumor type.

## Materials and Methods

All patient specimens were used with the permission of the Institutional Review Board at the University of Southern California. Patient data were de-identified prior to analysis.

### Analysis of data from The Cancer Genome Atlas

For analysis of epidemiological trends, ethnicity and patient age data for 210 TCGA EC cases (EAC G1, N = 93; USC G3, N = 97, MT-EC, N = 20) were included [8]. Two-tailed Fisher’s Exact tests were performed to examine differences between groups. For analysis of differentially expressed genes between EAC G1 and USC G3, we downloaded endometrial cancer RNAseq v2 data for 551 patients from TCGA. Among them, 92 cases were endometrioid endometrial adenocarcinoma Grade 1, and 94 were serous endometrial adenocarcinoma Grade 3. The R package DESeq2 [9] were used to analyze differential gene expression between these two groups, and an adjusted p-value<0.05 and fold change >±2 were used as filtering criteria.

### Sample selection and RNA extraction

We selected cases of formalin fixed, paraffin embedded endometrial cancers from the pathology archives at Los Angeles Country Hospital and USC Keck School of Medicine. All patient specimens were used with the permission of the Institutional Review Board at the University of Southern California. Patient data were de-identified prior to analysis. Each case was reviewed by an expert Gynecologic Oncology Pathologist (PMF) to confirm the histologic diagnosis. For MT-ECs, only cases with >50% of any one of the two components (Type I or Type II) were included. Tumor regions were marked on the hematoxylin and eosin stained slides and macrodissected (with a needle) from serial unstained sections. For mixed cases the different histological regions were isolated separately. Eight 10 μm unstained sections were cut from the tumor blocks. The tumor scraped off the slides and the RNA was extracted using the RecoverAll Total Nucleic Acid Isolation Kit for FFPE (Life Technologies), according to manufacturers instructions.

### Gene expression analysis

Reverse transcription was performed using 2μg of total RNA with random hexamers and the SuperScript III First Strand Synthesis SuperMix (Life Technologies). The cDNA was diluted to a final concentration of 10ng/mL. Custom Taqman Gene Expression Array Cards (Life Technologies) were desgned to detect 63 genes, in triplicate (including 3 endogenous controls). 200ng of cDNA was used per fill reservoir and the micro fluidic cards were run on the ABI OpenArray Real-Time PCR System (Life Technologies). Gene expression data were analyzed in ‘R’ using the ‘HTqPCR’ package [10].

### Immunohistochemistry

Immunohistochemistry was performed at the USC clinical pathology laboratory, using standard procedures. Briefly, 4 μm sections from MT-EC cases were deparaffinized and washed with ethanol. 3% H_2_O_2_ was applied to cooled sections to quench endogenous peroxidase activity. Tissues were blocked using a serum-free protein block (Dakocytomation). Sections were treated with an buffered saline solution, microwaved for 20 min, and incubated with a monoclonal anti-p16 antibody (Vantana) for 1 h at room temperature and the diaminobenzidine complex was used as a chromogen. Staiing was performed using the Leica automated Bond III instrument (Leica), and positive and negative control slides (incubated with secondary antibody only) were stained simultaneously. We tested for correlation between p16 gene and protein expression using simple linear regression performed in SAS 9.4.

## Results

### Epidemiology of mixed-type endometrial cancer cases

Serous and endometrial tumors differ in their epidemiological characteristics, with African American women and older women showing higher incidences of serous tumors, and Type I low-grade endometrioid tumors being the predominant tumor type in younger white women [11–14]. We used data for 210 endometrial cancers from The Cancer Genome Atlas (TCGA) project [8] to determine whether the epidemiology of mixed tumors most closely mimics that of serous or of endometrioid cases. In the dataset from TCGA, most endometrial adenocarcinoma (EAC) tumors occurred in white women, who represented 79.6% of EAC cases and 62.9% of USC cases. In constrast African American women represented just 9.7% of EAC cases but 25% of USC cases (P = 0.006, Table 1) as expected [11, 12]. African American women also accounted for 25% of MT-EC cases (**Table 1).** There were insufficient numbers of cases to detect a significant difference in frequencies between mixed and endometrioid cases (P = 0.1237) although the trend was similar to that for USC.

**Table 1:** Epidemiology of ECs (ethnicity). Statistically significant associations are indicated in bold, analyses compared the proportion of EAC, USC and MT-ECs that occur in White or African American Women. Other ethnic groups were excluded from the analyses. EAC G1 and USC were used as comparator samples, as indicated.

Group (N)	African American N(%)	White N (%)	P-Value (v EAC G1)	P-Value (v USC G3)
EAC G1 (93)	9 (9.7)	74 (79.6)		
USC G3 (96)	24 (25.0)	60 (62.5)	**0.006**	
MT-EC (20)	5 (25.0)	13 (65.0)	0.1237	1.000

Endometrioid cases also tend to occur in younger women, with 55.9% of cases occurring in women over 60 in contrast to 86.6% of serous G3 cases occurring in women older than 60 (P<0.0001). Mixed tumors also tend to occur in older women, with 80% of cases occurring in women older than 60 **(Table 2**), again this trend did not reach statistical significance due to the limited number of mixed cases.

**Table 2:** Epidemiology of ECs (age). Statistically significant associations are indicated in bold. EAC G1 and USC were used as comparator samples, as indicated, yo, years old.

Group (N)	<60 yo N (%)	>60 yo N (%)	P-Value (v EAC G1)	P-Value (v USC G3)
EAC G1 (93)	41 (44.1)	52 (55.9)		
USC G3 (96)	12 (12.5)	84 (87.5)	**<0.0001**	
MT-EC (20)	4 (20.0)	16 (80.0)	0.076	0.4736

### Analysis of genes differentially expressed between EAC and USC

We hypothesized that biomarkers that distinguish low grade endometrioid and high-grade serous tumors could give insight into the molecular phenotype and cellular origins of mixed tumors. To identify genes differentially expressed by low grade endometrioid and high-grade serous tumors we leveraged data from The Cancer Genome Atlas [8] and our own previously microarray data for endometrial cancer subtypes [15]**.** We downloaded endometrial cancer RNA-sequencing data for 92 Grade 1 endometrioid endometrial adenocarcinoma cases, and 94 Grade 3 serous endometrial adenocarcinoma cancer cases and identified the most differentially expressed genes. 3475 genes are significantly differentially expressed in these two groups using an adjusted P-value<0.5 and ±2 fold change as filtering criteria. 1979 genes were upregulated and 1494 genes downregulated in USC G3 compared to EAC G1. The most significantly differentially expressed genes are listed in **Table 3**.

**Table 3:** The most significantly differentially expressed genes between EAC G1 and USC G3. Performed using data from The Cancer Genome Atlas, listed in order of log2 fold change. The top 10 upregulated and downregulated genes are shown. Log_2_ FC, log2 fold-change in gene expression for USC G3 relative to EAC G1; SE, standard error; BH, Benjamini & Hochberg adjusted p-values. ‘Mean’ denotes mean expression for each group based as normalized RNAseq reads.

Gene Name	Log2 FC	SE	P-value	BH Adjusted P-value	EAC G1 Mean	USC G3 Mean
***Low in USC***						
*SERPINA11*	-6.07	0.37	2.6X10^-61^	3.5X10^-58^	236.3	3.8
*MT4*	-5.75	0.74	7.3X10^-15^	8.9X10^-14^	49.8	1.0
*BPIL1*	-5.73	0.41	2.5X10^-45^	8.0X10^-43^	556.8	3.2
*GP2*	-5.64	0.55	5.5X10^-25^	2.2X10^-23^	908.8	8.8
*IHH*	-5.61	0.31	4.3X10^-74^	1.1X10^-70^	3145.0	74.9
*DKK4*	-5.44	0.42	1.3X10^-37^	2.0X10^-35^	5220.9	39.5
*C1orf64*	-5.43	0.31	3.1X10^-69^	5.7X10^-66^	476.6	13.9
*TFF3*	-5.33	0.28	2.6X10^-83^	1.8X10^-79^	23559.6	688.8
*FGF20*	-5.30	0.42	5.3X10^-36^	6.6X10^-34^	72.1	2.1
*SCGB2A2*	-5.05	0.34	1.5X10^-50^	9.0X10^-48^	1955.2	6.4
**High in USC**						
*MAGEC2*	6.03	0.61	3.1X10^-23^	1.0X10^-21^	0.1	79.0
*L1CAM*	5.64	0.25	3.8X10^-111^	7.6X10^-107^	51.1	3922.3
*CLDN6*	5.41	0.29	3.6X10^-80^	1.5X10^-76^	34.9	4031.4
*XAGE2*	5.25	0.47	5.2X10^-29^	3.0X10^-27^	3.0	71.7
*MAGEA4*	5.14	0.53	3.9X10^-22^	1.1X10^-20^	3.3	366.6
*EYA4*	5.04	0.35	3.5X10-48	1.7X10^-45^	13.7	178.6
*FOXI3*	5.03	0.45	1.3X10-28	7.0X10-27	0.7	16.9
*GFRA4*	5.00	0.52	1.4X10-21	3.6X10-20	1.2	30.5
*CDH18*	4.76	0.40	6.2X10-33	5.4X10-31	6.9	91.2
*GAGE1*	4.76	0.59	6.8X10-16	9.1X10-15	1.0	167.9

We selected 60 biomarkers based on differential expression in endometrial G1 and serous G3 tumors (**S1 Table**), 20% of these biomarkers had also previously been identified in our previous gene expression microarray of endometrial cancer subtypes [15]. We also included 3 candidate biomarkers selected from the literature: *IMP3*, a marker commonly expressed in USC but not in EAC [16] as well as *PAX8* and *MUC16*, biomarkers commonly expressed in high-grade serous ovarian cancer (a tumor type that shares many molecular and histological characteristics with USC) [8, 17]. In addition, PAX8 is overexpressed in serous uterine carcinoma compared to endometrioid adenocarcinoma [18].

### Analysis of candidate gene expression in mixed and pure endometrial cancers

We extracted RNA from formalin-fixed paraffin embedded cases of pure serous (N = 5), pure endometrioid (N = 5), and mixed-type (N = 5) endometrial carcinomas. Patients and samples information are listed in **Table 4**.

**Table 4:** Patient information for samples used in this study. W, white; H, Hispanic.

Patient Number	Tumor Type	Race	Patient Age (years)	Sample No	Histology (%)	FIGO Grade
1	Mixed	W	63	1	EAC (60%)	G1
				2	USC (40%)	G3
2	Mixed	W	71	3	EAC (70%)	G1
				4	USC (30%)	G3
3	Mixed	H	66	5	EAC (50%)	G1
				6	USC (50%)	G3
4	Mixed	W	61	7	EAC (50%)	G1
				8	USC (50%)	G3
5	Mixed	W	68	9	EAC (40%)	G1
				10	USC (60%)	G3
6	Pure	H	37	11	EAC (100%)	G1
7	Pure	H	40	12	EAC (100%)	G2
8	Pure	H	64	13	EAC (100%)	G1
9	Pure	H	40	14	EAC (100%)	G2
10	Pure	W	47	15	EAC (100%)	G2
11	Pure	H	56	16	USC (100%)	G3
12	Pure	H	60	17	USC (100%)	G3
13	Pure	H	63	18	USC (100%)	G3
14	Pure	H	61	19	USC (100%)	G3
15	Pure	W	60	20	USC (100%)	G3

**Fig 1:**
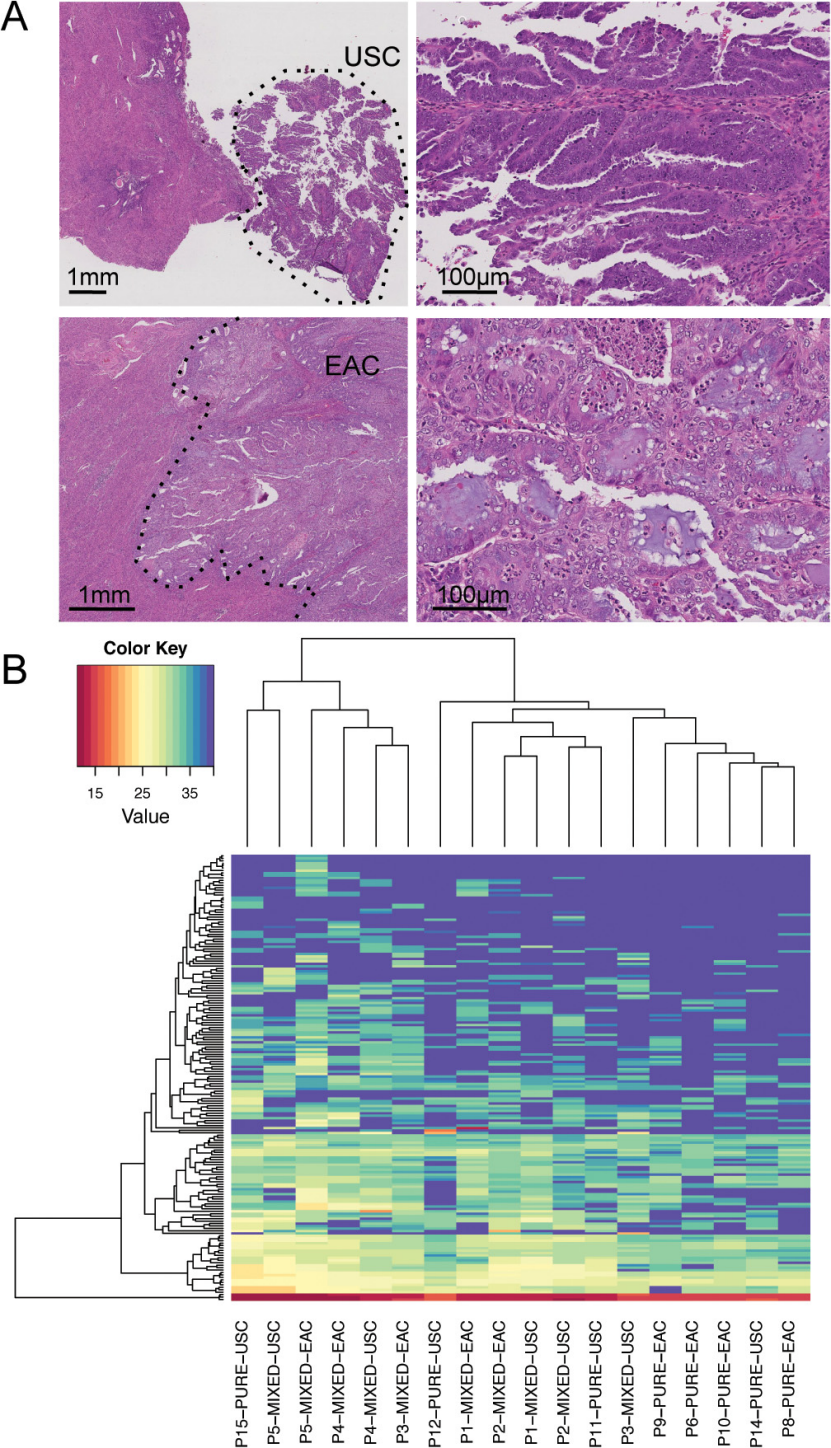
Molecular analysis of pure and mixed macrodissected endometrial cancers. (A). Gene expression profiling of MT-EC. H&E stained sections of USC and EAC region within an MT-EC (Patient 5). Low power images are shown on the left, the dashed line indicates the region macrodissected for molecular analyses. Representative high power images of the respective histologies are shown on the right. (B) Gene expression profiling of MT-EC. Unsupervised hierarchical clustering of macrodissected pure and mixed EC cases, based on the expression of a panel of 60 candidate genes. Pure EAC cases tend to cluster together; similary pure USC cases cluster together. Macrodissected regions of mixed cases also cluster together, with mixed cases more closely related to pure USCs.

For mixed cases, RNA was isolated from EAC and USC components separately (**Fig 1A**); the percentage of the serous component ranged from 30–60%. We analyzed expression of the 60 candidate genes using custom TaqMan microfluidic gene expression array cards. Two cases (one pure USC case and one pure EAC case) showed overall higher Ct values (i.e. lower gene expression) compared to the remaining samples and thus were excluded from the subsequent analyses. Unsupervised clustering suggested mixed and pure cases tend to cluster separately, but USC cases interspersed throughout the mixed cases suggests that pure USC cases are more closely related to mixed cases compared to pure EAC cases (**Fig 1B**).

**Fig 2:**
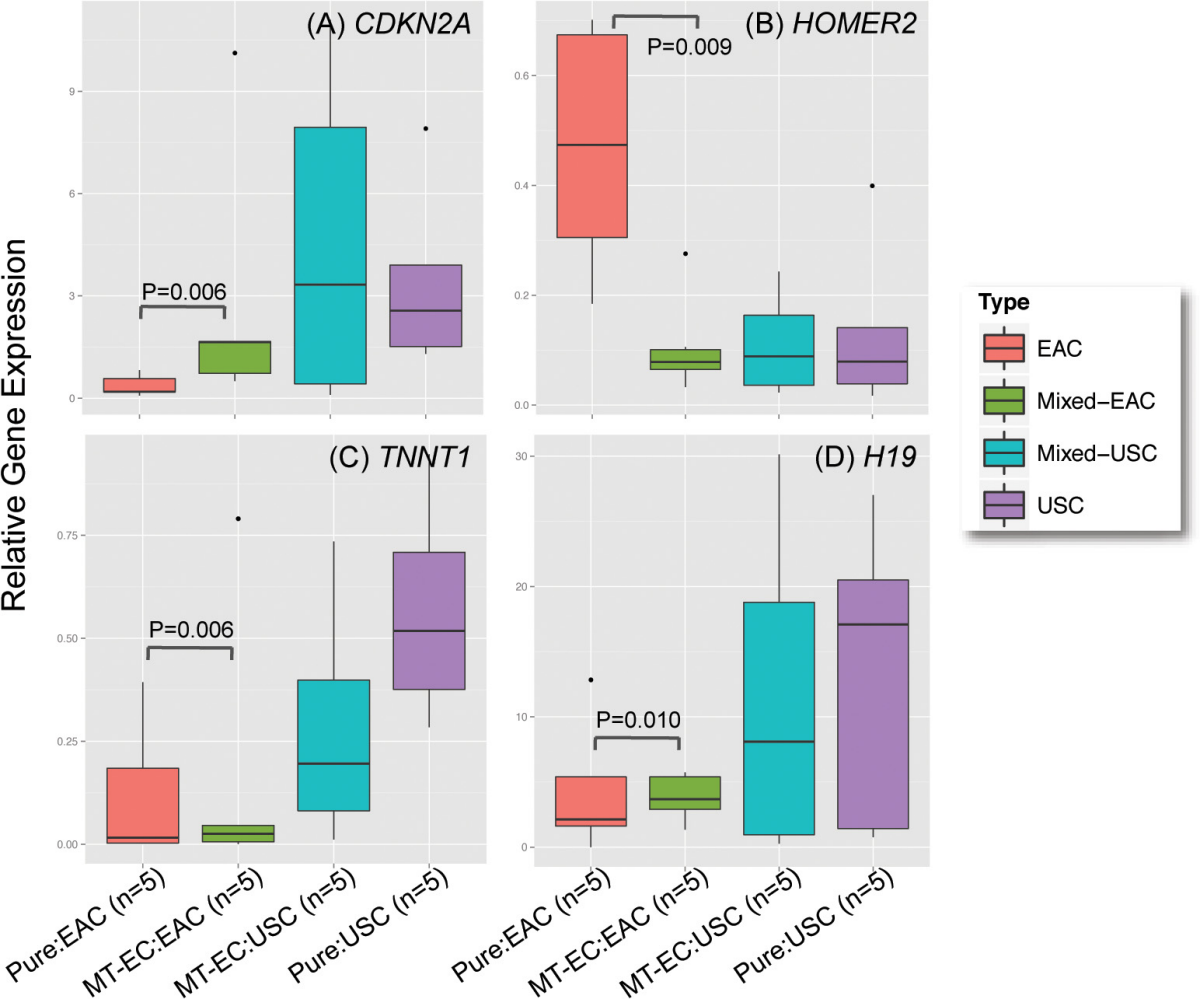
Genes differentially expressed between pure EAC and EAC regions within MT-ECs. Normalized relative gene expression, the horizontal line indicates median expression, the box indicates the 25^th^ and 75^th^ percentiles of the data and dots represent outlier datapoints.

**Fig 3:**
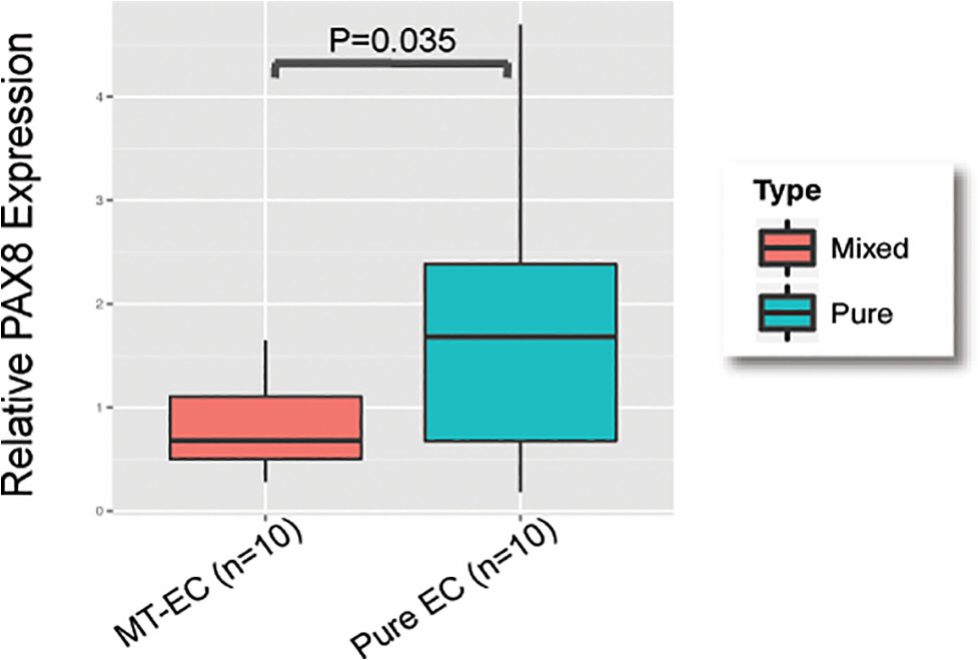
*PAX8* is differentially expressed between pure and mixed-type tumors. Normalized relative gene expression, the horizontal line indicates median expression, the box indicates the 25^th^ and 75^th^ percentiles of the data and dots represent outlier datapoints.

We performed a differential gene expression analysis to identify genes differentially expressed between pure and mixed tumors. We performed pair-wise comparisons of USC and EAC components in mixed tumors compared to pure tumors of the same histology. There were no genes differentially expressed between USC regions in mixed tumors compared to pure USC cases but four genes were significantly differentially expressed when mixed EACs were compared to pure EACs (**Fig 2**). These genes were *CDKN2A* (P = 0.006, fold change (FC) = 16.65), *HOMER2* (P = 0.009, FC = 0.15), *TNNT1* (P = 0.006, FC = 39.99) and *H19* (P = 0.010, FC = 10.48). When all pure tumors were compared to all mixed tumors, pure tumors exhibited higher expression of *PAX8* (P = 0.035), but no other genes were significantly differentially expressed (**Fig 3**). In addition, there were no differentially expressed genes when we compared EAC vs USC component within the mixed tumors.

**Fig 4:**
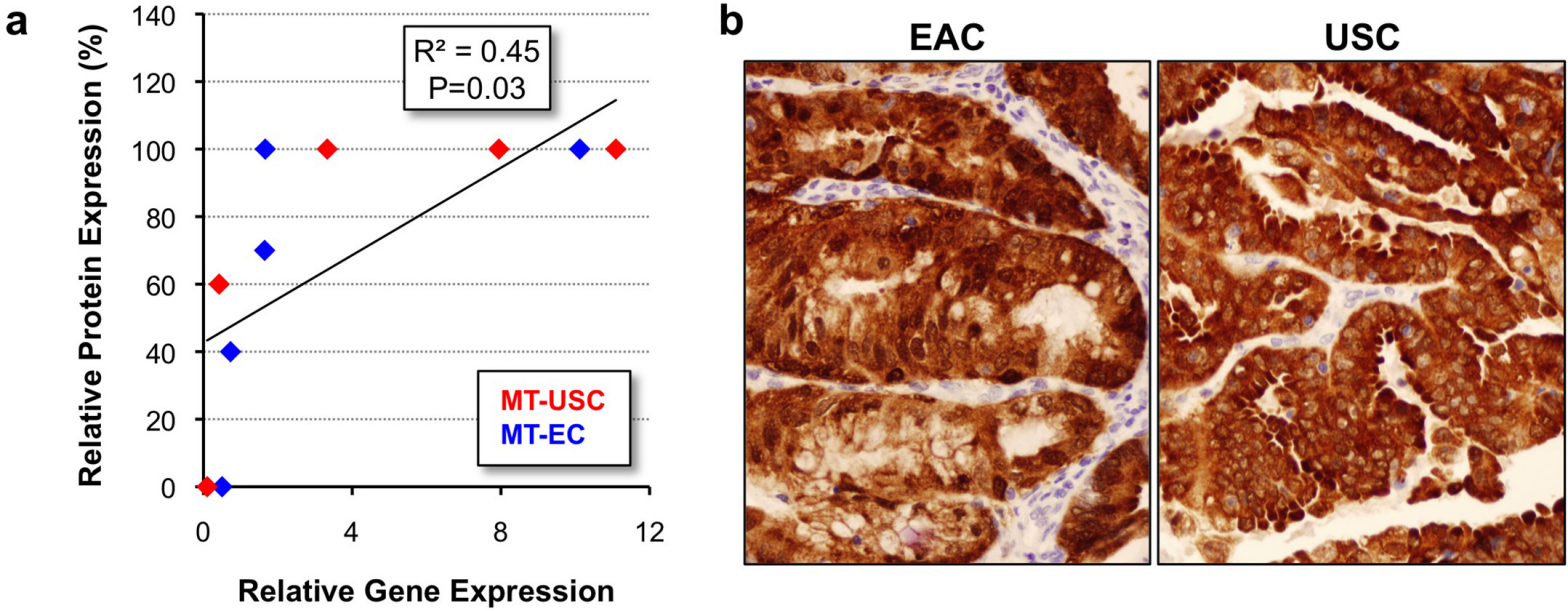
Comparison of p16 gene and protein expression. (A) Linear regression shows p16 gene and protein expression are highly correlated in USC and EAC components of MT-ECs. Protein expression is denoted at the percentage of cells staining positive plotted against relative, normalized gene expression. (B) P16 protein expression in USC and EAC components of a mixed tumor. 400X magnification.

Expression of p16 (*CDKN2A*) was validated using immunohistochemistry. There was a significant positive relationship between p16 gene and protein expression (R = 0.45, P = 0.03, Fig 4A). An example of a mixed tumor exhibiting high expression of p16 in both the EAC and USC regions can be found in Fig 4B.

## Discussion

Misdiagnosis of mixed-type endometrial carcinoma (MT-EC) is a significant source of morbidity and mortality for EC patients, and so the identification of patients with a high-grade component is of critical importance. Patients with advanced-stage EC do far worse than those with localized disease, with survival rates of only 17.5% for patients with late-stage disease. This is even worse than for epithelial ovarian cancer, a disease considered to be highly lethal, where patients with advanced-stage disease have 5-year survival rates of 27.4% [1]. There is no disputing the fact that there is an urgent need for improved diagnosis and treatment of endometrial cancer patients with the most aggressive disease subtypes. However, diagnosis of cases with a high-grade component of sometimes less than 10% is particularly challenging, and there are currently no biomarkers that can be used to accurately identify MT-ECs. Clinical studies of prognoses of patients with MT-ECs have yielded contradictory results: some report that MT-ECs with a serous component have the same prognoses as pure USC cases [2, 19], whereas other studies suggest MT-ECs have more favorable outcomes than their pure serous counterparts [20]. Moreover the biology of MT-ECs is poorly understood, and it is not known whether these tumors are truly mixed, or if the predominant histology represented indicates the true histology and etiology of the tumor.

One challenge for molecular analysis of MT-EC is the lack of fresh specimens for genomic research, which has no doubt hindered genome-wide profiling efforts. We therefore macrodissected formalin-fixed paraffin embedded MT-EC specimens to profile the Type I and Type II components separately to avoid the signal from one component masking the signal from the second component. The main finding of our study was the molecular similarity between USC and MT-ECs. Whereas pure USC tumors and USC regions within MT-ECs showed no significant differences in gene expression, four markers were significantly differentially expressed between pure EACs and EAC regions within a mixed tumor. Interesting, the most differentially expressed gene, *CDKN2A*, encodes the p16 gene involved in cell cycle progression, and is a known marker of USC [21]. This suggests that regions within mixed tumors that histologically resemble EAC exhibit molecular profiles more reminiscent of USC. The other genes differentially expressed in mixed versus pure EC tissues have not been previously implicated in endometrial cancer. *TNNT1* encodes a protein subunit of troponin, which is involved in the contraction of striated muscle. It is not clear how this gene could be involved in endometrial cancer etiology although *TNNT1* is overexpressed in metastatic uterine leiomyosarcoma, which suggests *TNNT1* is associated with more aggressive tumor cell phenotypes. *H19* is an imprinted non-protein coding gene located on chromosome 11, in close proximity to *IGF2*. *H19* has a well established role in the development of multiple cancer types, including breast, prostate and ovarian [22–25]. *H19* has not been well studied in endometrial cancer, but our data suggest *H19* may be a novel marker for the more aggressive endometrial tumor subtypes. Finally *HOMER* was highly expressed in pure EAC cases but expressed at low levels in pure USCs as well as EAC and USC areas within MT-ECs. Very little is known about the function of *HOMER2*. *HOMER2* is significantly hypermethylated in colorectal adenomas [26] and may be involved in anchorage-independent growth [27]. Our data suggest *HOMER2* may be involved in the development of low-grade EAC but not MT-EC or USC.

The similarity of MT-ECs to USC was also suggested from an epidemiological perspective, albeit to a lesser extent. Although endometrial cancer is less common in African American women, this ethnic group are more likely to develop USC and have poorer prognoses [11]. There was a trend for MT-ECs to occur in African American women more than would be expected by chance, although this study was underpowered to identify a statistically significant association. MT-ECs also tended to occur in older women, in the age group where USC is more common. Larger studies will be required to validate these preliminary observations, but it appears that the epidemiology of MT-ECs more closely resembles that of USC than EAC.

One caveat of these analyses is that the case with the lowest Type II component consisted of 30% USC and so in the present analyses we could not test whether tumors with a Type II component as low as 5% show molecular similarities with USC. While we expect that all MT-ECs with USC components will show similar molecular profiles, larger studies will be required to validate these findings in different populations and in tumors with a wider range of Type II component.

One limitation of our approach was the lack of global transcriptomic data for MT-ECs for the discovery phase of the study. Our approach was based on the rationale that MT-ECs with EAC or USC components would show molecular similarities to tumors of pure histology of the representative subtypes. However it is also plausible that novel MT-EC specific biomarkers will exist; and it is notable that the top biomarker identified as differentially expressed between pure and mixed cases was *PAX8*, a candidate gene included because of the common expression of this biomarker in high-grade serous and clear cell ovarian cancers [28]. Recently we also found PAX8 overexpression was associated with the serous subtype of endometrial cancer as well as EC tumor grade [18], yet unexpectedly, in the present study PAX8 expression was low in MT-ECs compared to pure EC counterparts. This suggests that while mixed tumors are highly similar to pure USC tumors, there are some molecular differences that exist, and that mixed tumors represent a *bone fide* separate subgroup, albeit with some molecular and epidemiological similarities to pure USC. The functional significance of low PAX8 expression in MT-ECs is unclear, but our data suggest that PAX8 is not required for the development of this tumor type.

This study reinforces the idea that a high-grade component indicates a more aggressive case of endometrial cancer, and suggests that even mixed cases that are predominantly EAC will behave like pure USC thus highlighting the importance of identifying any patient with a high-grade component in their endometrial tumors. Our epidemiological findings suggest that tumors from older patients and patients of African-American ancestry with EAC should undergo more extensive sampling for diagnostics to ensure no high-grade component is present. Since *PAX8* is commonly expressed in pure EC cases but is low/absent in MT-ECs, it is possible that the absence of PAX8 expression could be of utility for the identification of mixed cases, conversely, analysis of *CDKN2A*, *H19* or *TNNT1* expression could be used to identify cases with Type II components. Global analyses of MT-ECs will be an essential next step in the characterization of this tumor subtype to ensure further accuracy of patient diagnoses and treatment.

## Supporting Information

S1 Table Selected candidate genes arrayed on microfluidic cards. FC, fold change for USC G3 relative to EAC G1); MA, was this gene also identified in microarrays from Fauceglia *et al*. [15].
(XLSX)
